# Prognostic Performance of ATT and mGCS Scores in Dogs and Cats with Traumatic Injury

**DOI:** 10.3390/vetsci12111081

**Published:** 2025-11-13

**Authors:** Avital Neimann, Tomer Weingram, Martin Kožár

**Affiliations:** 1 Clinic of Small Animals, University of Veterinary Medicine and Pharmacy in Košice, Komenského 73, 041 81 Košice, Slovakia; 2 Vet Center, Hameretz St. 7, Rosh-HaAyin 4801707, Israel; tomer.weingram@gmail.com

**Keywords:** polytrauma, veterinary emergency and critical care, Animal Trauma Triage (ATT), modified Glasgow Coma Scale (mGCS), prognosis, mortality, multiple organ dysfunction syndrome (MODS)

## Abstract

Trauma is a major cause of morbidity and mortality in dogs and cats. While prognostic tools are well-established in human medicine, few guidelines exist in veterinary trauma care. The Animal Trauma Triage (ATT) score and modified Glasgow Coma Scale (mGCS) are used to assess illness severity, but their clinical utility in veterinary patients remains undervalued. This study aimed to evaluate the prognostic value of ATT and mGCS scores and their association with organ dysfunction and survival in polytraumatized veterinary patients. We hypothesized that multi-organ failure (MOF) is more prevalent in non-survivors and correlates with higher ATT and lower mGCS scores. A prospective observational study was conducted for 30 patients (20 dogs and 10 cats) admitted to two veterinary hospitals. Clinical data, trauma scores, and outcomes were collected and analyzed. The overall survival rate was 83.3%; blunt trauma accounted for 80% of cases. Non-survivors (*n* = 5) had higher respiratory rates at admission (*p* = 0.01). The ATT score accurately predicted all fatalities, while the mGCS score showed limited prognostic value. MOF was the leading cause of death in 60% of non-survivors. ATT appears to be a more reliable tool for outcome prediction, enabling improved triage, resource allocation, and early intervention in veterinary trauma cases.

## Simple Summary

A major cause of morbidity and mortality in dogs and cats is traumatic injuries, but tools to predict outcome in veterinary patients remain limited. This prospective study investigated the prognostic performance of two commonly used clinical scoring systems—the Animal Trauma Triage (ATT) score and the modified Glasgow Coma Scale (mGCS)—in 30 dogs and cats with polytrauma. The results showed that the ATT score was a highly accurate predictor of survival, correctly identifying all non-survivors based on a score threshold. In contrast, the mGCS score showed good but less consistent accuracy, with one non-survivor scoring in the normal range. The main cause of death among non-survivors was multi-organ dysfunction syndrome (MODS), emphasizing the importance of early recognition and intervention. These findings support the use of the ATT score as a reliable tool for assessing injury severity and guiding treatment decisions in veterinary emergency settings.

## 1. Introduction

Trauma is defined as any sudden “wound or tissue injury” caused by external forces, including animal–animal interactions such as penetrating bite wounds, human–animal interactions such as blunt trauma from motor vehicle accidents or other causes such as falls from height [1]. The severity of trauma ranges from mild to life-threatening and, in some cases, fatal.

In small animals, trauma is a leading cause of morbidity and mortality and represents one of the most common reasons for admission to emergency care [1]. Polytrauma, defined as a clinically significant injury that affects multiple organ systems or body regions, poses a particular challenge due to its potential to destabilize patient physiology and the frequent need for intensive hospitalization [2,3].

The primary traumatic injury causes direct tissue damage, which is often irreversible. However, the secondary injury cascade, which includes systemic inflammatory responses, ischemia–reperfusion injury, and metabolic disturbances, frequently proves to be more decisive in determining clinical outcome [4]. This cascade may culminate in multiple organ dysfunction syndrome (MODS), a critical and often fatal complication [5].

Veterinary management begins with a ’triage’: a rapid assessment that typically lasts 30 to 60 s and prioritizes patients according to severity. The triage progresses in structured steps. The primary survey assesses respiratory, cardiovascular, neurologic, and urinary systems, with stabilization guided by the principles of airway, breathing, and circulation (ABC) and by rapid evaluation of the major body systems (MBSs). The secondary survey consists of a comprehensive examination from the nose to the tail, with particular attention given to musculoskeletal injuries. In human medicine, a tertiary survey is frequently performed to re-evaluate previous findings and detect previously unrecognized injuries [3]. Shock is a frequent consequence of severe trauma and is defined as peripheral circulatory failure in which oxygen delivery does not meet tissue demand. This results in a shift from aerobic to anaerobic metabolism, with rapid energy depletion, lactic acidosis, and progressive hypoxia [3,6,7]. Compensatory mechanisms, including tachycardia, vasoconstriction, redistribution of blood flow to vital organs, reduced urine output, and increased oxygen extraction, may temporarily stabilize the patient. When these fail, cell death and organ failure follow [3]. Poor prognostic indicators in veterinary patients include traumatic brain injury, skull fractures, recumbency, hematochezia, acute respiratory distress syndrome (ARDS), disseminated intravascular coagulation (DIC), pneumonia, positive pressure ventilation (PPV), and the use of vasopressors [7].

Diagnosis and stabilization require a combination of laboratory analysis and imaging. Early laboratory tests include packed cell volume and total protein, followed by hematocrit, glucose, and calcium, each of which provides prognostic information [3,6]. Imaging is essential for rapid evaluation, with radiography and ultrasonography being the most commonly used modalities to assess pulmonary contusions, pneumothorax, diaphragmatic hernia, free fluid and organ displacement. Point-of-care ultrasound (POCUS), ECG, and blood gas analysis further aid in rapid assessment. Advanced imaging techniques such as computed tomography and magnetic resonance imaging are valuable but are generally reserved for later stages when patients are stable enough for anesthesia.

Although veterinary trauma care differs from human medicine in many respects, there are parallels. Small animal patients resemble human trauma victims in rural settings, where blunt injuries are common, delays in discovery and transport are frequent, and structured pre-hospital triage systems are limited [8]. These factors contribute to higher mortality. Nevertheless, human trauma care principles, particularly structured triage, resuscitation strategies, and severity scoring systems, can be adapted to veterinary medicine when feasible.

Trauma scoring systems, among the assessment tools available, provide an objective framework to classify injury severity and forecast clinical outcome. In veterinary practice, the two most widely used are the modified Glasgow Coma Scale (mGCS), adapted from human medicine to assess neurologic injury [9], and the Animal Trauma Triage (ATT) score, developed specifically for small animals [10]. Both scoring systems quantify trauma severity, have been shown to correlate with mortality, and assist in guiding clinical decision-making, while their relationship with the risk of MODS remains less clearly established [11,12].

The mGCS introduced by Shores in 2001 to evaluate the prognosis in animals with TBI [13] evaluates motor activity, brainstem reflexes, and level of consciousness, and each category is scored from 1 (severely abnormal) to 6 (normal), producing scores of 3 to 18. The prognosis is classified according to the score ranges: 15–18 (Good), 9–14 (Guarded), and 3–8 (Grave). Lower scores indicate more severe neurologic dysfunction and poorer survival, with scores of 8 or less associated with a survival rate of 50% [9], and each one-point increase in the score is associated with a 2.06-fold increase in the probability of survival [12,14].

The ATT score was first developed by Rockar in 1994 to predict the outcome in polytraumatized veterinary patients [10]. The score assesses six body systems: perfusion, cardiac, respiratory, eye/muscle/integument, skeletal, and neurologic. Each category is scored from 0 (slight or no injury) to 3 (indicating severe injury), contributing equally to a total score ranging from 0 to 18. Higher scores reflect a greater severity of the injury and an increased risk of mortality and systemic complications [6,15]. An ATT score of 9 has been associated with an estimated 50% probability of survival [6,15], and each additional point has been associated with a 15% higher risk of developing systemic inflammatory response syndrome (SIRS) in vehicular trauma [16], as well as significantly higher odds of mortality; 1.78 in cats and 2.07 in dogs [14].

Both the ATT score and the mGCS have demonstrated utility in retrospective studies, although prospective validation remains limited. Large-scale registry data confirm the excellent discriminative performance of the ATT score in dogs (AUROC = 0.92) and its moderate performance in cats (AUROC ≈ 0.75) [14]. Analysis of 3599 polytraumatized dogs confirmed that each additional ATT point approximately doubles the mortality risk (OR ≈ 2.07; AUROC 0.92, 95% CI 0.91–0.94) [11]. In cats, a registry study of 1065 cases of bite wounds showed that increasing the quantiles of the ATT score resulted in a 4.5-fold increase in the odds of death, while surgical intervention reduced the risk of mortality by 84% [14,16]. Additionally, in 25 cats with high-rise trauma, an ATT cutoff ≥ 6 predicted non-survival with 75% sensitivity and 90% specificity (AUROC = 0.917), while each one-point reduction in mGCS was associated with a 2.41-fold increase in odds of death [17]. Blunt trauma in cats was also associated with lower mGCS and higher ATT scores, which are correlated with poorer survival compared to penetrating trauma [11].

Thus, recent feline studies provide quantitative measures of predictive performance in different types of trauma. In bite wound cases, each one-point reduction in mGCS doubles the odds of mortality (AUROC ≈ 0.78). Craniofacial trauma studies show similarly that lower mGCS predicted a poorer outcome, with AUROC values ranging from 0.74 to 0.81 [13,18]. Data in canine patients remain comparatively limited, with only registry-level analyses offering a broader perspective [19]. This highlights the importance of reporting canine trauma cases in which scoring and outcome can be evaluated, allowing inter-species comparison and further refining the clinical utility of ATT and mGCS for early prognosis, triage, and resource allocation in veterinary trauma patients.

This study prospectively evaluates the prognostic value of the ATT and mGCS scores in polytraumatized dogs and cats, hypothesizing that higher ATT and lower mGCS scores are associated with greater organ dysfunction and increased mortality, thus aiding early outcome prediction and clinical decision-making.

## 2. Materials and Methods

### 2.1. Study Design and Population

A prospective, observational, multi-center study was conducted over a 13-month period (September 2019 to October 2020) in the emergency and critical care units of the University of Veterinary Medicine and Pharmacy in Košice, Slovakia, and the Vet Center in Herzliya, Israel. A total of 30 patients (20 dogs, 10 cats) with varying degrees of polytraumatic injuries were enrolled. Inclusion in this study required medical history and clinical signs consistent with polytrauma that affects multiple body systems. Only patients with complete medical records from admission to outcome were included.

### 2.2. Data Collection

Upon admission, data was recorded for each patient, including signalment (species, breed, age, sex, weight, reproductive status), trauma history (type, cause, injury location), and findings of physical examination. Initial vital signs, including rectal temperature (RT), heart rate (HR), respiratory rate (RR), and capillary refill time (CRT), were documented. Concurrent diseases were recorded.

### 2.3. Trauma Scoring

ATT and mGCS scores were calculated for each patient based on the findings of the initial physical examination. The ATT score was calculated by assigning a score of 0 (no/slight injury) to 3 (severe injury) for each of the six categories (perfusion, cardiac, respiratory, eye/muscle/integument, skeletal, and neurologic), up to a total possible score of 18 [10]. The mGCS was calculated by assessing three categories (motor activity, brainstem reflexes, and level of consciousness), each scoring from 1 (severe dysfunction) to 6 (normal), for a total possible score of 3 to 18 [9].

### 2.4. Laboratory Analysis and Diagnostic Imaging

Blood samples (2–3 mL) were collected by venipuncture (Cephalic, Saphenous or Jugular vein) upon admission, prior to or shortly after the initiation of fluid resuscitation. Complete blood count (CBC) and serum biochemistry profiles were performed. The analyzers used included the IDEXX ProCyte Dx and Catalyst One (IDEXX Laboratories Inc., Westbrook, ME, USA) at the University of Veterinary Medicine and Pharmacy in Košice (UVLF) and the VetScan^®^ HM5 and VS2 (Abaxis Inc., Union City, CA, USA) at Vet Center Hospital. The key parameters evaluated were hematocrit (HCT), platelet count (PLT), total protein (TP), glucose (Glu), blood urea nitrogen (BUN), creatinine (CREA), and electrolytes, assessing hemodynamic, cardiovascular, and renal systems. The reference range of blood parameters was recorded according to the blood analysis machines used in each hospital, and units were converted as needed.

### 2.5. Advanced Treatment

The treatment variables were divided into medical or surgical intervention, including recording the medications and surgical procedures performed. The need for specific advanced treatment modalities such as hypertonic saline, endotracheal intubation, administration of mannitol, corticosteroids, benzodiazepines, phenobarbital, or oxygen supplementation was recorded.

### 2.6. Outcome Assessment and Statistical Analysis

The primary outcome was recorded as survival or non-survival to hospital discharge. Non-survival was further classified as death despite treatment or euthanasia (due to grave prognosis, financial constraints, or both). Multi-organ dysfunction was identified as the cause of death based on clinical progression and terminal laboratory findings consistent with the failure of two or more organ systems. All statistical analyses were performed using the Jamovi software (Version 1.2; The Jamovi Project, Sydney, Australia; available at https://www.jamovi.org). Continuous variables were assessed for normality using the Shapiro–Wilk test. Normally distributed data are expressed as mean ± standard deviation (SD), whereas non-normally distributed data are presented as median and interquartile range (IQR). Differences between survivors and non-survivors were analyzed using the independent *t*-test or the Mann–Whitney U test, as appropriate. Categorical variables were compared using the Chi-square (*χ*^2^) or Fisher’s exact test. A *p* < 0.05 was considered statistically significant. Logistic regression and receiver operating characteristic (ROC) curve analyses were performed to evaluate the predictive performance of trauma scores (ATT and mGCS).

## 3. Results

### 3.1. Study Population Characteristics

A total of 30 patients (20 dogs, 10 cats) were included in the study. The cohort consisted of half males and half females. The median age was 1.85 years (ranging from 2 months to 9 years) and the mean body weight was 7.75 kg (ranging from 850 g to almost 28 kg). The majority of patients admitted to the UVLF veterinary hospital were purebred canines and felines of different breeds, while the majority of patients admitted to Vet-Center were mixed breeds.

Blunt trauma was the most common type of injury, accounting for 80% (*n* = 24) of cases, 53.3% of dogs and 30% of cats, with motor vehicle accidents as the leading cause (66.7%, *n* = 20). Penetrating trauma, including bite wounds, accounted for 16.7% (*n* = 5) of cases, 13.3% in dogs and 3.3% in cats. Other causes were a fall from height (3.3%, *n* = 1) or an unknown trauma (10%, *n* = 3). A summary of the demographics of the population and types of trauma is provided in Table 1.

### 3.2. Clinical Findings, Laboratory Findings and Diagnostics

Initial laboratory values indicated that many admitted dogs were anemic; however, this condition was uncommon in cats. Treatment records showed that no dogs or cats at the UVLF veterinary hospital received blood products to treat anemia, while three dogs in Vet-Center received blood product transfusions. Other laboratory findings showed that many polytraumatic patients were in a hyperglycemic state.

Diagnostic imaging included abdominal POCUS (*n* = 17/57%, *n* = 5/16.7%), thoracic POCUS (*n* = 9/30%, *n* = 3/10%) and X-rays (*n* = 18/60%, *n* = 10/33%), in canines and felines, respectively. Surgical procedures were performed on nine (55%) dogs and seven (70%) cats, as seen in Table 2. Eight patients with polytrauma (26.7%) had undergone centesis; two abdominocentesis, five thoracocentesis, and one cystocentesis. Centesis was used for both diagnostic and therapeutic reasons. In the 16 patients who underwent surgery, the procedures included orthopedic (*n* = 6, 37.5%), hernia repair (*n* = 3, 18.7%), nasal, thoracic, esophageal tube or urinary catheter placements (*n* = 7, 43.7%), mesh repair of liver bleeding (*n* = 1, 6.25%), enucleation (*n* = 1, 6.25%) and debridement (*n* = 2, 12.5%).

Patients with evidence of polytrauma had injuries in multiple areas of the body, including the abdomen, thorax, extremities, or the head. Six (20%) patients had pelvic fractures, while another six had long bone fractures, predominantly the femur. Almost all fractures were of more than one pelvic bone or multi-fragmented femoral fractures. Four out of 30 study patients (13.3%) presented traumatic brain injury. One dog (5%) presented with a proptosed ocular sphere, with hematoma of the upper and lower eyelid, blood in the anterior chamber, ruptured eyeball and retinal detachment, treated by enucleation. Two patients had non-clinically significant ocular injuries. Pulmonary contusions, pneumothorax, and body herniation (diaphragmatic or inguinal) were diagnosed in 10 (33.33%), 11 (36.7%) and 3 (10%) patients, respectively. Other notable conditions included hypovolemic shock (*n* = 4/13.3%). Physical injuries observed were tail necrosis (*n* = 1/3.33%), instability of the right tarsus joint (possible ligament rupture and brachial plexus avulsion with dorsoflexion) (*n* = 1/3.33%), extensive hemorrhages with bilateral subcutaneous emphysema (*n* = 1/3.33%), crepitation and carpus dislocation (*n* = 1/3.33%), and one patient presented with free fluid around the spleen and liver as the sole injury, managed medically.

### 3.3. Trauma Score and Outcome

The overall survival rate to hospital discharge was 83.3% (25/30). A comparison of clinical parameters and trauma scores between survivors and non-survivors is presented in Table 3. No statistical significance was found between survivors and non-survivors in HR, RT, HCT, TP, Glu, PLT, UREA, NA, or K values. Nevertheless, no differences were found between the use of medical or surgical treatments between the groups. Advanced treatment modalities (e.g., hypertonic saline, the use of oxygen and specific medications) were used in similar numbers in both survivors and non-survivors, with no statistical significance. However, a higher tendency to use intubation was found in the non-survivor group compared to the survivor group, although it did not reach statistical significance. Similarly, higher creatinine levels and lower rectal temperature were recorded in the non-survivor’ group, but the numbers did not reach statistical significance. Hypovolemic shock, commonly seen in polytrauma, may explain the lower temperature. The median respiratory rate was found to be significantly higher in non-survivors compared to survivors, with 78 and 100 breaths/minute in the non-survivors group, of dogs and cats, respectively, compared to 32 and 44 in the survivors group (*p* = 0.015, large Effect size).

Non-survivors had lower mGCS scores (median 9 vs. 18) and higher ATT scores (median 6 vs. 10), with specificity and sensitivity for each, 100 and 80% for mGCS and 100 and 60% for ATT, with a threshold of 10 points, similar to that used in previous studies [9]. A lower threshold for the ATT score of ≥9 had a higher sensitivity (75%), with a high specificity remaining. A higher threshold for mgrs of ≤11 has a sensitivity of 100%, but a lower specificity.

**Figure 1:**
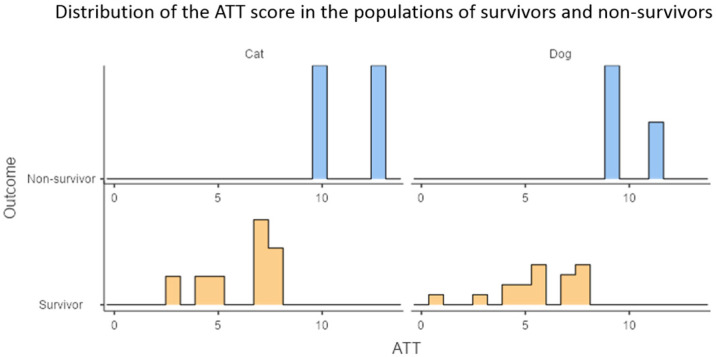
Distribution of Animal Trauma Triage (ATT) scores among surviving (blue) and non-surviving (orange) dogs (right) and cats (left) with polytraumatic injuries. *Y*-axis represents the number of patients (*n*), and the *X*-axis represents the prognostic score (as points). Both populations showed a correlation between higher ATT scores and non-survival, with the ATT score demonstrating perfect discrimination for survival outcome.

**Figure 2:**
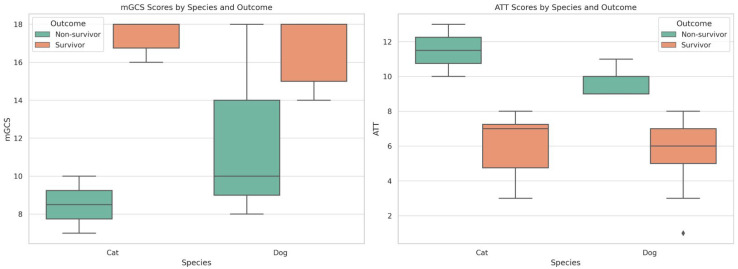
Distribution of mGCS and ATT scores by species and survival outcome. Across species, non-survivors had markedly lower mGCS scores and higher ATT scores compared with survivors, with complete separation of ATT scores between outcome groups. Boxes represent interquartile ranges, the horizontal line within each box indicates the median, whiskers show the minimum and maximum values, and diamonds (♦) indicate outlier values.

The median ATT score for all patients in the polytraumatic study was seven, with median values for dogs and cats: 6.5 and 7, respectively (range 1–13). Most of the non-survivors were young female dogs. No surviving patient had a score ≥ 9, and no non-surviving patient scored < 9. The ATT score showed high overall precision in both groups, with all survivors scoring ≤ 8, and all non-survivors scoring ≥ 9, representing a low probability of survival. The ATT score showed similar precision in both dog and cat populations, as demonstrated in Figure 1 and Figure 2.

**Figure 3:**
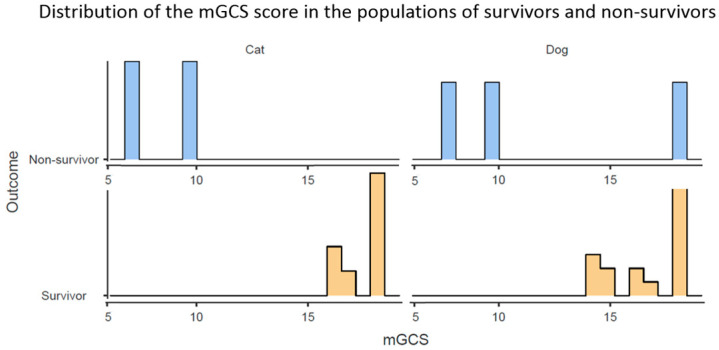
Distribution of modified Glasgow Coma Scale (mGCS) scores among surviving (blue) and non-surviving (orange) dogs (right) and cats (left) with polytraumatic injuries. *Y*-axis represents the number of patients (*n*), and the *X*-axis represents the prognostic score (as points). Both populations of dogs and cats showed a correlation between lower mGCS scores and non-survival, with the mGCS score showing lower accuracy in the non-survivor group, but high accuracy in the survivor group.

The median mGCS for all patients in the polytraumatic study was 17.5 (range 7–18); no animal scored < 7. The mGCS score showed an overall lower precision in the non-survivor group, with one patient scoring the highest score (seen in Figure 2 and Figure 3). However, in the survivor group, the prediction ability of mGCS showed high accuracy.

**Figure 4:**
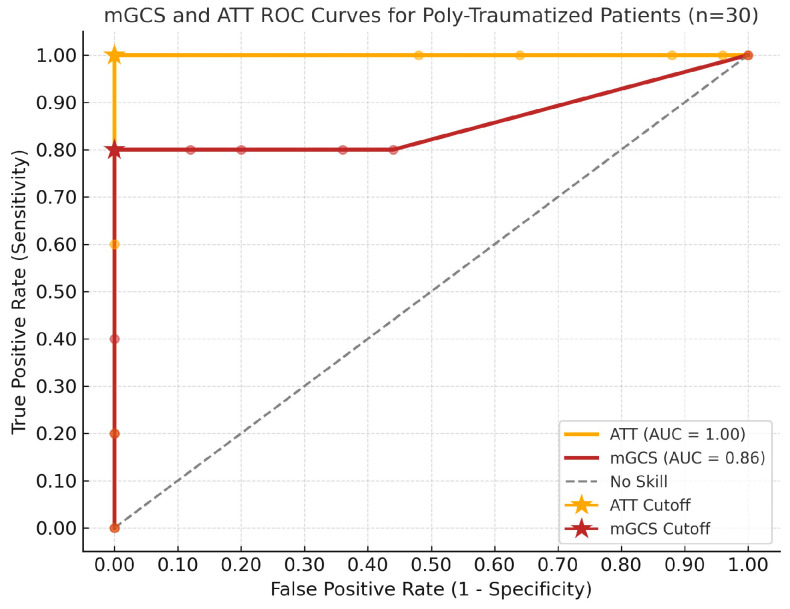
Receiver operating characteristic (ROC) Curves for Animal trauma triage score (ATT) and Modified Glasgow Coma Scale (mGCS) scores in a population of 30 polytraumatic injured dogs and cats. Stars illustrate the optimal cut-off sensitivity and specificity for predicting non-survival.

The discriminative ability of the scores showed that mGCS was a strong predictor of survival, but not a perfect classifier of survival outcome, with an AUROC of 0.86 (95% CI 0.53–1), while the ATT score showed a perfect discriminative ability, with an AUROC of 1.0, as shown in Figure 4.

Of the five non-survivors, three died despite treatment, and two were euthanized due to a grave prognosis. One patient was euthanized for both grave prognosis and financial reasons. The cause of death for three of the five non-survivors (60%) was attributed to MODS and cardiopulmonary collapse. All non-survivors had an ATT score of ≥8, and four had an mGCS score of ≤14, which are thresholds previously defined as predictive of non-survival in veterinary trauma patients [9,12,14,15]. A summary of non-survivor cases is provided in Table 4.

## 4. Discussion

Frequently, polytraumatized dogs and cats in this study population exhibited clinical signs consistent with shock and shock-associated metabolic derangements, including metabolic acidosis and hyperglycemia. Hyperglycemia arises not only from the catecholamine-driven stress response but also from cortisol release, inflammatory cytokine activity, and insulin resistance, particularly pronounced in patients with traumatic brain injury, and has been consistently linked to a worse prognosis and reduced survival [3,6,9]. Lactic acidosis develops when inadequate tissue perfusion occurs, forcing a shift from aerobic to anaerobic metabolism, resulting in impaired ATP production, accumulation of lactate, and progressive cellular hypoxia. This disturbance reflects the severity of the shock and has been associated with a poor outcome in traumatized veterinary patients [3,6,7].

In veterinary trauma patients, the ATT score has been shown to predict short-term outcome and the need for intensive care in both dogs and cats suffering from traumatic injuries [1,10]. Recent feline-focused studies have further demonstrated the prognostic value of both ATT and mGCS in specific trauma contexts, including high-rise falls, craniofacial injuries, and bite wounds, underscoring their clinical utility in cats [13,17,18].

In this prospective multi-center study population, the prognostic utility of ATT and mGCS scores was evaluated in 30 polytraumatized dogs and cats, to identify patients prone to develop MODS through the trauma cascade, allowing rapid treatment and optimized resource allocation to prevent fatal outcomes. Our findings contribute to canine-specific data, which remain limited compared to feline cohorts where there are larger-scale prognostic studies. This cross-species perspective highlights important similarities but also underscores that predictive accuracy may vary depending on the type of injury and neurological involvement.

The prevalence of blunt trauma, particularly motor vehicle accidents, as the main cause of injury aligns with established epidemiology in small animal trauma [6], and MODS was the leading cause of death, consistent with previous reports [3]. Interestingly, feline studies report similar injury distributions, with falls and blunt trauma dominating, while head trauma is more prevalent in cats than in dogs [18]. This epidemiological overlap supports the use of comparable scoring tools, while emphasizing the need to validate thresholds for each species.

The overall survival rate of 83.3% observed in this study is consistent with previous reports [12,20]; however, some studies have documented higher mortality rates [8,21]. Lower mortality rates of 7.6% and 7.3% have been reported in studies with substantially larger populations—nearly 8000 dogs and cats—which limits direct comparison [11]. Nevertheless, the 83.3% survival rate remains broadly consistent with survival rates of 88% to 92% reported in larger retrospective cohorts [1,11]. Notably, feline-specific studies have shown survival rates exceeding 90% in certain trauma subtypes, particularly high-rise falls, where the prognosis is generally favorable [17]. The slightly lower survival in our mixed cohort likely reflects the greater heterogeneity and severity of injuries, particularly MODS-driven mortality.

All cats were accurately classified according to their trauma scores, ATT, and mGCS. However, one dog was not correctly predicted: its ATT score of 9 classified it as a non-survivor, whereas its mGCS score of 18 suggested survival. This discrepancy likely reflects the strength of mGCS to predict the outcome in neurologically affected patients, but a lower reliability in diverse polytrauma cases without central nervous system involvement.

The ATT score demonstrated excellent precision in predicting the outcome and, in most cases, was correlated with the presence of MODS or organ dysfunction, which is associated with increased mortality risk. This underscores the value of trauma severity scores in identifying severely affected patients and supports their use as part of the overall clinical evaluation of dogs and cats with polytrauma.

Furthermore, this study population was highly heterogeneous, including patients with a wide range of injury severities. Inclusion of patients with relatively minor injuries can influence results, as the scoring system was designed primarily to identify the most critically injured individuals prone to progressing into the trauma cascade leading to SIRS, MODS, and death. Nevertheless, the ATT score exhibited perfect discrimination in predicting non-survival, with a median ATT score of 7 for the entire cohort, indicating that minor injuries did not significantly affect the results. The low discrimination observed in the Eye/Muscle/Integument category may reflect its broad nature, encompassing injuries from minor abrasions to severe trauma, failing to distinguish injuries that do not impact the prognosis.

A key limitation of this study was the small sample size and the exclusion of patients with incomplete admission data, which may have influenced the interpretation of the score. The mGCS demonstrated good discriminative ability (AUROC = 0.86), outperforming a random classifier, but the wide 95% confidence interval (0.53–1.0) reflects the limited number of non-survivors and the small cohort size. Trauma patients are highly dynamic, with rapidly changing physiological parameters affecting the ATT score and potentially altering the outcome predictions over short periods. Although mGCS correctly identified four of the five non-survivors (80%) in neurologically affected patients, its predictive precision was lower in those without neurological dysfunction. Its labor-intensive nature may also limit practical utility in emergency settings, potentially delaying treatment and under-recognizing severely affected patients. These factors highlight the need for a cautious interpretation of trauma scores and further validation in larger study populations.

Additionally, score precision may be affected by physiological compensation mechanisms, which can mask the true severity of the injury, as well as by the experience of the clinician performing the assessments; more experienced personnel may detect clinical signs with better sensitivity, thus improving predictive reliability.

Despite these limitations, our study highlights the critical prognostic value of trauma scores in predicting organ dysfunction. Sixty percent of non-survivors succumbed to complications consistent with MODS and circulatory collapse, emphasizing that, although initial physical trauma triggers a cascade, the systemic response and development of organ failure are often the ultimate determinants of mortality. Continuous monitoring beyond initial triage, focusing on early detection of respiratory, cardiovascular, and renal impairment, remains essential.

Beyond trauma scores, the only statistically significant finding was a higher respiratory rate among non-survivors. This may reflect compensatory mechanisms in response to hypoxia, metabolic acidosis, pain, or severe involvement of the central nervous system, such as high cervical spinal cord trauma. Other parameters not evaluated in this study—blood lactate, the need for PPV, and SpO_2_ at admission—may also have a prognostic value, which warrants further investigation.

In clinical practice, trauma scores are valuable components of initial patient assessment but relying on them as the sole predictor may be misleading, potentially underestimating injury severity and occult shock. The ATT score, given its strong predictive performance, should be integrated into a comprehensive evaluation that includes a thorough physical examination, continuous monitoring of vital signs, and targeted diagnostics. The primary value of these scores lies in the rapid identification of high-risk patients requiring immediate intervention and close monitoring.

Emerging feline literature supports these findings, demonstrating high predictive precision of ATT cutoff points (≥6 or ≥10) for mortality, particularly in high-rise falls, bite wounds and craniofacial trauma [13,17,18]. Although large-scale validation similar to that is limited in dogs, registry-level studies suggest comparable trends [19], highlighting the need for species-specific thresholds and further prospective canine research. In this study population, each one-point increase in the ATT score greater than eight was associated with approximately a two-fold increase in the odds of mortality (OR ≈ 2.07 in dogs and 2.02 in cats), while each 1-point decrease in mGCS below 14 similarly doubled the risk of death (OR ≈ 2.02 in dogs and 2.04 in cats), similar to previous reports [9,11,14]. This study demonstrates that both dogs and cats follow a similar pattern, in which each one-point change in ATT (increase) or mGCS (decrease) approximately doubles the odds of death, with thresholds of ATT ≥ 9 and mGCS ≤ 14 corresponding to a 50% probability of survival. Each one-point increase in ATT was associated with a 5.9-fold increase in MODS odds, with a sensitivity of 100% and a specificity of 93% (AUC 0.98). Conversely, a one-point reduction in mGCS corresponded to a 1.4-fold higher likelihood of MODS, with a sensitivity of 67%, specificity of 93%, and an AUC of 0.73. These findings suggest that ATT outperforms mGCS in predicting the risk of MODS in polytraumatized dogs and cats.

To the authors’ knowledge, this is the first prospective multicenter study to evaluate both dogs and cats with polytrauma using ATT and mGCS scores, linking these tools not only to survival, but also to the risk of multi-organ dysfunction. Notably, discrepancies observed between the predictions of ATT and mGCS in neurologically intact versus neurologically compromised patients underscore the practical limitations of mGCS in heterogeneous polytrauma populations, extending previous neuro-focused studies [9,14] and complementing larger registry-based evaluations [11,12].

Integration of automated scoring systems could improve the clinical utility of trauma scores by dynamically updating ATT in real time as physiological parameters—temperature, respiratory rate, heart rate—change, allowing more responsive and efficient decision-making. Future research should explore the combination of trauma scores with prognostic biomarkers, such as lactate and excess base, to improve predictive precision in both canine and feline populations [13,17].

## 5. Conclusions

In this prospective study of polytraumatized dogs and cats, the ATT score demonstrated high precision as a standalone predictor of mortality. The development of multi-organ dysfunction syndrome was a major contributor to death in non-surviving patients. Although trauma severity scores are indispensable in emergency triage, some indicators, such as the mGCS, should be used as part of a comprehensive patient assessment rather than as a sole prognostic indicator due to their high but not perfect predictive ability. Further research involving larger and more severely affected populations is warranted to better characterize the relationship between initial trauma scores and the development of specific organ dysfunctions. Nonetheless, these results highlight the importance of the ATT score, which exhibited perfect discrimination in predicting non-survival, enabling rapid identification of patients at increased risk of developing MODS and facilitating prompt and effective treatment to minimize delays in diagnosis and care.

## Figures and Tables

**Table 1:** Distribution of type and cause of trauma by species and survival outcome (*n* = 30).

		Survivors (83.3%)		Non-Survivors (16.7%)	
Type of Trauma	Cause of Injury	Canine (*n* = 17)	Feline (*n* = 8)	Canine (*n* = 3)	Feline (*n* = 2)
**Blunt**	Motor Vehicle Accident (HBC)	12 (70.6%)	4 (50.0%)	2 (66.7%)	–
	High-rise Syndrome (HRS)	–	1 (12.5%)	–	1 (50.0%)
	Other	1 (5.9%)	2 (25.0%)	–	–
**Penetrating**	Bite wounds	4 (23.5%)	1 (12.5%)	1 (33.3%)	1 (50.0%)

Distribution of trauma type and cause of injury among survivors and non-survivors, stratified by species. Most canine and feline patients sustained blunt trauma, primarily due to motor vehicle accidents (HBC), while penetrating injuries (bite wounds) accounted for a smaller proportion of cases.

**Table 2:** Advanced treatment modalities used in the study population of polytraumatized patients and outcome. LRS, Ringer lactate solution; ETT, Endotracheal tube; ICU, intensive care unit.

Species (% Total)	Canine 20 (66.7%)	Feline 10 (33.3%)
Surgical procedure performed	9 (30%)	7 (23.3%)
Mannitol administered?	2 (6.7%)	1 (3.33%)
Hypertonic saline administered?	2 (6.7%)	1 (3.33%)
Isotonic saline/LRS	19 (63.3%)	9 (30%)
Oxygen supplementation required?	10 (33.3%)	5 (16.7%)
ETT performed?	8 (26.7%)	3 (10%)
Phenobarbital administered?	8 (26.7%)	3 (10%)
Benzodiazepines administered?	3 (10%)	1 (3.33%)
Presence of seizure activity?	2 (6.7%)	1 (3.33%)
Glucocorticoid administered?	3 (10%)	1 (3.33%)
Undergone orthopedic procedure?	5 (16.7%)	3 (10%)
Undergone emergency surgery?	4 (13.3%)	2 (6.7%)
Admission to ICU?	20 (66.7%)	10 (33.3%)
Received blood products transfusion?	3 (10%)	0 (0%)
**Outcome**		
Survived to discharge	17 (56.7%)	8 (26.7%)
Died despite treatment	2 (6.7%)	1 (3.33%)
Euthanized	1 (3.33%)	1 (3.33%)
**Euthanized—reason**		
Grave prognosis	0 (0%)	1 (3.33%)
Financial limitation	0 (0%)	0 (0%)
Both	1 (3.33%)	0 (0%)

**Table 3:** Clinical and laboratory variables recorded upon hospital admission for surviving and non-surviving dogs and cats with polytraumatic injuries.

Variable	Survivors (*n* = 25)		Non-Survivors (*n* = 5)	
	Canine (*n* = 17)	Feline (*n* = 8)	Canine (*n* = 3)	Feline (*n* = 2)
**RT (°C)**	38.0 (37.2–39.0)	38.6 (37.3–40.5)	38.2 (36.5–38.6)	37.3 (37.1–37.5)
**HR (bpm)**	140 (120–180)	200 (140–250)	104 (102–108) †	140 (120–160) †
**RR (bpm)** *	32 (20–60)	44 (20–78)	76 (72–100) †	100 (80–120) †
**HCT (%)**	42.8 (24.8–58.6)	32.7 (20.8–45.0)	29.3 (25.0–46.5)	29.3 (25.0–46.5)
**mGCS Score** *	18 (14–18)	16 (16–18)	10.6 (8–10.8)	8.5 (7–10)
**ATT Score** *	6 (1–8)	7 (4–8)	9 (9–11)	11.5 (10–13)

Comparison of selected clinical and laboratory variables between survivors and non-survivors. Variables are reported as median (interquartile range, IQR) for skewed data or mean ± SD for normally distributed variables. RT, Rectal temperature. RR, respiratory rate. HR, Heart rate. HCT, hematocrit value. MGCS, Modified Glasgow Coma Scale. ATT, Animal Trauma Triage. * *p* value < 0.05 indicates a significant difference between survivors and non-survivors. Statistically significant results were *p* = 0.0015 for mGCS, 0.0008 for ATT score, and 0.01 for RR, using the Mann–Whitney U test. Effect size (*r*) for mGCS is 0.49 (moderate–large) and 0.7 for the ATT score (large). † For HR and RR in non-survivors; values are reported as median (range).

**Table 4:** Details of the non-survivor patients (*n* = 5).

Case ID	Species	Trauma Type	mGCS	ATT	Key Injuries	Outcome/Cause of Death
G-2351	Canine	Blunt (HBC)	8	9	Spinal fractures, quadriplegic	Euthanasia (due to grave prognosis)
H-1027	Canine	Penetrating (Bite)	18	9	Pneumothorax, suture dehiscence, wound necrosis, sepsis	Death despite treatment (cardiopulmonary failure during anesthesia, MODs)
H-1846	Canine	Blunt (HBC)	10	11	Multiple pelvic fractures, hip luxation, pneumothorax, paraplegic	Death (cardiopulmonary failure, MODs)
H-2244	Feline	Penetrating (Bite)	10	10	Pelvic bite wounds, loss of neurologic function	Euthanasia (due to grave prognosis)
3205	Feline	Blunt (HRS)	7	13	TBI (cleft palate, anisocoria, no PLR or menace), pneumothorax, carpal dislocation	Death (cardiopulmonary failure, MODs

## Data Availability

The original contributions presented in this study are included in the article. Further inquiries can be directed to the corresponding author.

## Abbreviations

The following abbreviations are used in this manuscript:

Abbreviation	Definition
ATT	Animal Trauma Triage
mGCS	Modified Glasgow Coma Scale
MODS	Multiple Organ Dysfunction Syndrome
TBI	Traumatic Brain Injury
CBC	Complete Blood Count
PCV	Packed Cell Volume
TP/TS	Total Protein/Total Solids
HCT	Hematocrit
HR	Heart Rate
RR	Respiratory Rate
CRT	Capillary Refill Time
USG	Ultrasonography
POCUS	Point-of-care ultrasound
PPV	Positive-Pressure Ventilation
DIC	Disseminated Intravascular Coagulation
ARDS	Acute Respiratory Distress Syndrome
SpO_2_	Peripheral Oxygen Saturation
ATP	Adenosine Triphosphate
BUN	Blood Urea Nitrogen
Glu	Glucose
ECG	Electrocardiography
SIRS	Systemic Inflammatory Response Syndrome
HBC	Hit by Car
